# Art therapy and emotional pain: a scoping review of physiological and biological measures

**DOI:** 10.3389/fnhum.2026.1736930

**Published:** 2026-03-11

**Authors:** Shokoufeh Moezzi, Olga Korostynska, Mimmu Rankanen, Haroon Khan, Parisa Gazerani

**Affiliations:** 1 ADEPT Research Group, Department of Mechanical, Electrical and Chemical Engineering, Faculty of Technology, Art and Design, OsloMet – Oslo Metropolitan University, Oslo, Norway; 2 Department of Art, Design and Drama, Faculty of Technology, Art and Design, OsloMet – Oslo Metropolitan University, Oslo, Norway; 3 Department of Life Sciences and Health, Faculty of Health Sciences, OsloMet – Oslo Metropolitan University, Oslo, Norway

**Keywords:** biological markers, neuroscience, pain, physiological measurements, visual art therapy

## Abstract

**Introduction:**

The increasing prevalence of mental health disorders and emotional pain poses a critical challenge to social well-being and healthcare equity. Visual art therapy is well established as a clinical and nonclinical intervention for emotional pain that promotes self-regulation and psychological insight. However, there is a lack of research that clearly maps the previous studies that use both subjective and objective measures to examine the impact of art therapy on emotional pain.

**Methods:**

This scoping review focuses on studies that use brain or physiological measurement in investigating the effect of art therapy on emotional pain in healthy adults. A systematic search of academic databases and scholarly information systems MEDLINE, PsycINFO, Engineering Village, Web of Science, Academic Search Ultimate, and Epistemonikos was conducted in May 2025. It identified 4,734 relevant records, of which 12 full texts were screened, and 6 studies met the inclusion criteria.

**Results:**

Evidence indicates that visual art therapy can improve mood and reduce stress, anxiety, fear, and sadness, also modulating activity across multiple brain regions. Overall, fNIRS studies reported increased activation in the left dorsolateral prefrontal cortex after art therapy, and studies on HR, skin conductance, salivary cortisol, sAA, IL-6, CRP, and RSA illustrated its positive effects in reducing stress, anxiety, and sad mood.

**Discussion:**

However, existing research has primarily addressed emotional pain, with no studies assessing its impact on physical pain in healthy populations using objective physiological or biological measures, showing that there is a gap for assessing physical pain improvement by art therapy. These findings highlight both the therapeutic potential of visual art interventions and the need for further research to explore their effects on physical pain.

**Systematic review registration:**

This review was registered on the Open Science Framework (https://osf.io/935kw, date created and registered: 24. 07. 2025).

## 1 Introduction

Pain, whether physical or psychological, is a complex and multidimensional experience that encompasses sensory, cognitive, and emotional components, and can significantly disrupt daily functioning and reduce quality of life (Melzack, 2001; Hohls et al., 2021). In this review, the term “emotional pain” refers to negatively valenced affective states such as stress, anxiety, sadness, fear, or distress, which are commonly operationalized in experimental and psychophysiological research using standardized induction paradigms and validated self-report or physiological measures. Moreover, in this review, emotional pain is used as an umbrella term for experimentally induced negative affective states that are commonly studied in psychophysiological and affective neuroscience research. Emotional pain is increasingly understood to share overlapping neural and biological mechanisms with physical pain, including activation of limbic and prefrontal circuits and stress-related biomarkers (Roseman, 2025).

One particularly underexplored aspect is emotional pain as a form of distress associated with experiences like grief, rejection, sadness, and trauma (Daly and Macchia, 2023; Estancial Fernandes et al., 2019). Unlike physical or chronic pain, emotional pain often lacks clear biological origins or visible symptoms, making it harder to assess, measure, and treat. It is, however, just as real and debilitating, with evidence suggesting it activates similar neural and inflammatory pathways as physical pain (Nan et al., 2021; Sturgeon and Zautra, 2016; Slavich et al., 2010). Despite its impact, emotional pain remains insufficiently addressed in current healthcare models. Emotional pain and disrupted emotion processing appear across multiple mental disorders, supporting their role as a shared underlying vulnerability, as almost half of individuals with a mental disorder also meet criteria for another disorder (1). Moreover, emotional distress and emotional pain are highly prevalent globally; in surveys of over 1.5 million adults across 113 countries, estimates of emotional distress (e.g., sadness, stress, worry) ranged from 25 to 31%, with increases over the past decade (2) (Piao et al., 2024). Furthermore, population-based evidence indicates that approximately one-third of individuals report significant emotional problems, which are associated with substantially lower health-related quality of life (3). Large-scale epidemiological evidence indicates that emotional stress is highly prevalent and worsening globally, with over half of the population affected in several countries and widespread declines in psychological well-being across age groups, living environments, and employment statuses (4). A recent study reported that 38% of Scottish adults experienced chronic pain in 2022 (Birtwistle et al., 2023). Another study conducted in 2023 reported that 21.5% of adults at Soshanguve Community Health Centre (CHC) in South Africa experienced chronic pain, which significantly affected mood (42.3%), relationships (47.9%), and enjoyment of life (39.4%) (Pandelani et al., 2023). The economic impact of living with pain is considerable. Drawing on data from two major Norwegian health surveys (HUNT3 and Tromsø6) researchers found that 36% of the 63,782 participants reported living with chronic pain. Over a six-year period (2010–2016), individuals with chronic pain incurred an average of €55,003 more in costs compared to those without pain. When scaled to the national level, these findings suggest that chronic pain contributes to an annual economic burden equivalent to 4% of Norway’s GDP. This study estimates that the societal cost of chronic pain may reach as high as €12 billion each year (Stubhaug et al., 2024). Beyond the financial implications, pain is a bio-psycho-social phenomena that deeply affects daily life: it decreases productivity, restricts social interactions, diminishes overall well-being, and leads to mental disorders such as depression and anxiety (Aaron et al., 2025). Its effects also extend to family members and caregivers, who often share the emotional and practical challenges (Lim and Zebrack, 2004; Dueñas et al., 2016). Despite advances in medicine and technology, pain remains a widespread and persistent problem (Breivik et al., 2006). For instance, while pharmacological treatments are commonly used, their undesirable side effects and limited short-term benefits underscore the need for effective non-pharmacological alternatives such as art therapy (Raymond et al., 2021; Gussak and Rosal, 2026). Given these realities, there is a growing demand for sustainable and holistic approaches to pain management (Shi and Wu, 2023). Arts therapies are increasingly recognized as practical and accessible approaches that target the multidirectional interplay between pain and its psychological and social dimensions, acknowledging that emotional and social factors can both result from pain and influence its intensity and persistence (Raudenská et al., 2023).

Art therapy, defined more specifically as visual arts therapy but may include also other modalities such as music, dance, and drama, is increasingly recognized as an effective intervention for alleviating physical and psychological pain and enhancing emotional well-being (Raudenská et al., 2023; Beerse et al., 2020; Haiblum-Itskovitch et al., 2018; Bugos et al., 2022). The formal concept of “art therapy” was first articulated in the 1940s by British artist Adrian Hill. While recovering from tuberculosis in a sanatorium, Hill discovered the therapeutic benefits of creating art. In his 1945 book “Art Versus Illness”, he detailed how engaging in artistic expression helped patients cope with their illnesses and emotional struggles (Hill, 1945). Hill is widely credited with coining the term “art therapy” and advocating for its use in clinical and mental health settings. His work inspired others, leading to the development of art therapy as a recognized field of study and practice. Notably, Margaret Naumburg was an early developer and she stressed more the importance of the art therapeutic relationship that combines art and therapist - not the healing power of art alone (Naumburg, 1987). In the present day, a wide spectrum of evidence-informed clinical and community-based art therapy practices have been developed and spread all around the world (Gussak and Rosal, 2026; Hills de Zárate et al., 2025).

This scoping review focusses on visual art therapy, an active form of art therapy that engages individuals in creative visual tasks such as drawing, doodling, and painting (Joschko et al., 2024). Through these hands-on activities, participants can express emotions, explore personal experiences, and support psychological healing in a non-verbal and reflective way, which has shown particular promise in addressing psychological distress (Haiblum-Itskovitch et al., 2018). Studies have shown that art-making helps older adults who suffer from chronic pain by helping them distract from their pain (Kim et al., 2023). Several studies highlight the effectiveness of visual art therapy in reducing emotional pain and related consequences of physical pain, such as depression and anxiety (Han B. et al., 2024). As these emotional states improve, the perception of physical pain may also change (Lumley et al., 2011). This suggests that by alleviating psychological comorbidities, visual art therapy may indirectly influence the perception of physical pain as well. Previous reviews have begun to explore the neurocognitive mechanisms underlying creative arts interventions, such as emotion regulation and activation of reward-related neural pathways (Barnett and Vasiu, 2024; Kaimal et al., 2017). However, these studies encompassed multiple art modalities, whereas our review focuses specifically on visual art therapy and its relationship to pain as measured by physiological outcomes. Evidence in this field remains limited and scattered. For example, a recent scoping review on paediatric pain interventions highlighted how heterogeneous and fragmented the available studies are, making it difficult to draw firm conclusions (Olaizola et al., 2024). Similar challenges are evident in the literature on visual art therapy and pain in adults. Unlike pharmacological treatments, visual art therapy is not associated with adverse side effects. Moreover, by promoting behavioral change and emotional resilience, it may offer long-term benefits. These characteristics position visual art therapy as a sustainable and holistic approach for individuals suffering from emotional pain.

Considering the current evidence, there is still a lack of understanding regarding the effect of visual art therapy on emotional pain in healthy individuals, as measured by physiological and biological biomarkers. By examining how visual art therapy modulates emotional pain, we aim to understand the underlying mechanisms of its effects. Insights gained from healthy participants can then inform the application of art therapy in other populations with different disorders, tailoring interventions to specific needs (Gruber and Oepen, 2018). In particular, research employing biological or physiological measures of emotional pain such as brain imaging, biomarker, heart rate, and cortisol, could provide stronger evidence of its effectiveness (Haiblum-Itskovitch et al., 2018; Zhang et al., 2021; Kaimal et al., 2016). Establishing such evidence is crucial, as it could influence policymakers and healthcare regulators to formally recognize visual art therapy as an effective treatment option. Demonstrating measurable improvements in both psychological outcomes (for example, reductions in depression and anxiety linked to physical pain) and overall quality of life could also highlight its potential to reduce healthcare costs by preventing the need for additional treatments (Sturgeon and Zautra, 2016; Slavich et al., 2010).

Art therapy is an innovative interdisciplinary intervention that has shown promise in supporting individuals experiencing emotional distress. By engaging individuals in creative expression, it facilitates emotional processing (Kret and Ploeger, 2015), psychological insight, and self-regulation. It is non-pharmacological and adaptable across age groups making it an inclusive and sustainable tool for pain and mental health management. However, while qualitative and observational studies support its benefits, the biological mechanisms behind its effects remain largely unexplored.

This scoping review aims to map and synthesize the existing literature on the impact of visual art therapy on emotional pain. In particular, it focuses on studies that incorporate both subjective reports and objective physiological measures, with the goal of clarifying current evidence, identifying research gaps, and informing future research on the integration of visual art therapy as a sustainable, evidence-informed approach to pain management.

## 2 Materials and methods

The Preferred Reporting Items for Systematic Reviews and Meta-Analyses Extension for Scoping Reviews (PRISMA-ScR) was followed for this review (Tricco et al., 2018). This review was registered on the Open Science Framework; Registration link: https://osf.io/935kw (date created and registered: 24.07.2025). Data were synthesized using a descriptive numerical summary and qualitative narrative synthesis, consistent with PRISMA-ScR guidance for scoping reviews to map and characterize existing evidence (Lockwood et al., 2019; Levac et al., 2010; Arksey and O'malley, 2005). No formal quality appraisal or meta-analytic synthesis was conducted, as the aim was to map existing evidence rather than evaluate intervention effectiveness.

### 2.1 Inclusion and exclusion criteria

Studies were eligible for inclusion if they involved healthy adult participants aged 18 to 60 years. In this review, healthy individuals refer to participants without diagnosed neurological or psychiatric disorders, although they may experience transient stress or emotional discomfort induced experimentally (Gierthmühlen et al., 2015). This age range was selected to reduce potential confounding factors, particularly those associated with age-related neurodegenerative risk, thereby ensuring greater validity and reliability of the findings. Studies involving children, animals, or individuals with neurological disorders (such as stroke or brain injury) were excluded. The review focuses on structured visual art therapy interventions, including drawing, painting, and sculpting. These interventions may be facilitated by a trained art therapist or implemented through guided instructions for independent art-making. Studies that did not include any form of active visual art therapy, or used other creative modalities like music, dance, or narrative-only therapies, were excluded. In this review, the term ‘art therapy’ refers to interventions delivered within a structured therapeutic framework led by trained professionals, whereas ‘art-making’ or ‘art-based activity’ reflects terminology used in the original studies when activities were not explicitly conducted as formal therapy.

To qualify, studies also needed to involve brain, physiological measurement tools, or biological biomarker such as Electroencephalography (EEG), functional Near Infrared Spectroscopy (fNIRS), Electrocardiography (ECG), Heart Rate (HR), Heart Rate Variability (HRV), and Salivary cortisol. Studies without any physiological or neurological measurement components were excluded. Additionally, the study had to include a pain component, either through experimental induction [using standardized and ethically approved laboratory paradigms such as fear-eliciting film clips or emotion recall tasks (Godinho et al., 2006)] or assessment of physical or emotional pain (before and after art therapy) and examine how art therapy affected that pain. Studies focusing solely on chronic pain without any measurement component were excluded because the focus was on studies that link art therapy to physiological responses, rather than those relying exclusively on self-report.

Eligible studies were required to report brain or physiological signals as a primary outcome and could additionally include other relevant outcomes such as pain perception or self-report (for example, Visual Analogue Scale (VAS), Numeric Rating Scale (NRS), McGill Pain Questionnaire) (Hawker et al., 2011), emotional or affective state (for example, State–Trait Anxiety Inventory) (Hallegraeff et al., 2020), or participant engagement. Studies that did not include measurable outcomes related to the interaction between art, the brain, and the body were excluded.

Only empirical studies (for example, quantitative, qualitative, mixed methods, or case studies) were included. Reviews, protocols, editorials, and theoretical papers were excluded from the main analysis, though they were considered for background context. Only English-language studies were included.

With two reviewers contributing to primary (title + abstract) screening, the reviewers agreed on defining any unclear cases as potentially relevant (i.e., apply a tag “maybe” during the title and abstract screening). The “maybe” tag for potentially relevant publications, while increasing the workload at the step of full-text screening, aimed to avoid false-negative exclusion.

### 2.2 Search strategy and study selection

**Figure 1:**
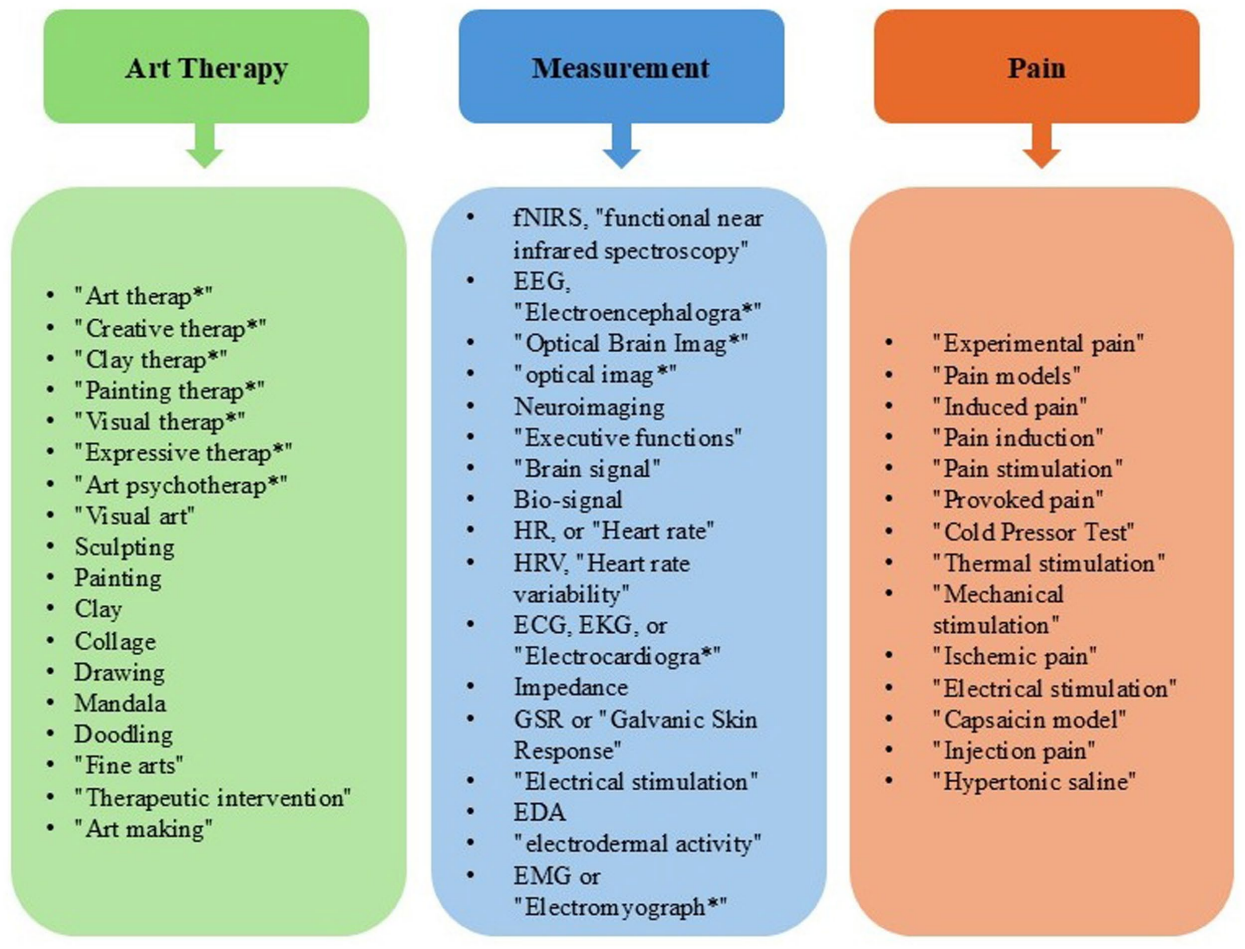
Keyword search.

The search strategy was established by a professionally trained librarian. Electronic searches were performed in the following databases: MEDLINE (Ovid) (*n* = 928), Psycinfo (*n* = 323), Engineering Village (*n* = 448), Web of Science (*n* = 5,906), Academic search ultimate (*n* = 513), and Epistemoniks (*n* = 125). Since Epistemonikos primarily indexes systematic reviews rather than primary research, it may yield few eligible studies directly. However, its inclusion was justified because it can help identify systematic reviews that reference relevant primary studies. Other databases, such as CINAHL and Embase, were excluded due to the specific focus of our review, which primarily targeted psychological and engineering perspectives on art therapy. No date limits were applied. Keywords included combinations of (“Art therap*” or “Creative therap*” or “Clay therap*” or “Painting therap*” or “Visual therap*” or “Expressive therap*” or “Art psychotherap*” or “Visual art” or Sculpting or Painting or Clay or Collage or Drawing or Mandala or Doodling or “Fine arts” or “Therapeutic intervention” or “Art making”) and (fNIRS or “functional near infrared spectroscopy” or EEG or “Electroencephalogra*” or “Optical Brain Imag*” or “optical imag*” or Neuroimaging or “Executive functions” or “Brain signal” or Bio-signal or “Heart rate” or HR or “Heart rate variability” or HRV or ECG or EKG or “Electrocardiogra*” or Impedance or GSR or “Galvanic Skin Response” or “Electrical stimulation” or EDA or “electrodermal activity” or EMG or “Electromyograph*”) and (“Experimental pain” or “Pain models” or “Induced pain” or “Pain induction” or “Pain stimulation” or “Provoked pain” or “Cold Pressor Test” or “Thermal stimulation” or “Mechanical stimulation” or “Ischemic pain” or “Electrical stimulation” or “Capsaicin model” or “Injection pain” or “Hypertonic saline”). Figure 1 illustrates the keywords search. A sample of our search strategy is given in Appendix A. Once all references were retrieved, a member of the research team uploaded the data to Rayyan^1^ (Ouzzani et al., 2016), a tool used to remove all duplicate articles and further manual screening (include, exclude, or maybe). A PRISMA flowchart diagram of the search and screening process was provided to demonstrate the articles that were selected for data extraction and those that were deemed ineligible, including the reasons for ineligibility. Using Rayyan, all articles were screened one-by-one using our inclusion and exclusion criteria divided amongst two team members (SM, PG), each article had to be independently approved by two screeners. When eligibility could not be confidently assessed during the title and abstract screening, the article was advanced to full-text review. If ambiguity persisted after full-text assessment, a third reviewer was invited to resolve the disagreement and reach a final decision. First, abstracts and titles alone were screened, then full texts, and finally those passing the full text stage were pulled for data extraction.

### 2.3 Data extraction

The data extraction form was jointly developed by the research team, including two reviewers, to determine relevant variables. Extracted data included: bibliographic info (author, year, country, journal), Population (sample size, demographics), Intervention (type, duration, delivery), Outcomes (pain type, measurement tools, physiological markers, emotional measures), Study design, Main findings, and Limitations.

## 3 Results

### 3.1 General overview

**Figure 2:**
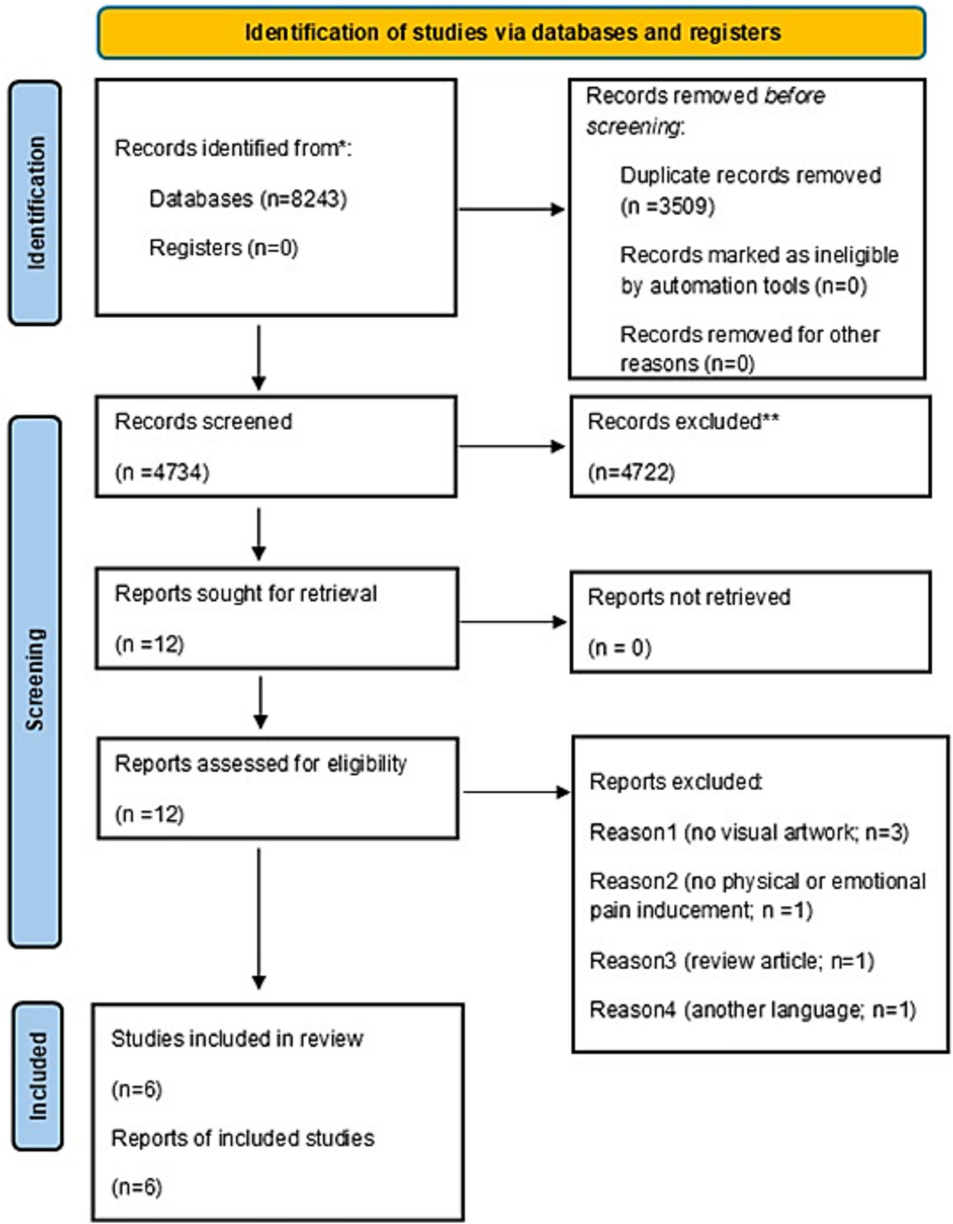
PRISMA flowchart of the screening process. *Electronic searches were performed in the following databases: MEDLINE (Ovid) (*n* = 928), PsycINFO (*n* = 323), Engineering Village (*n* = 448), Web of Science (*n* = 5,906), Academic search ultimate (*n* = 513), and Epistemoniks (*n* = 125). ** All records were screened and excluded manually by the reviewers; no automation tools were used during the screening process.

The search strategy yielded 8,243 studies (see Figure 2). Using Rayyan, 3,509 duplicates were removed, resulting in 4734 studies to be screened. After screening titles and abstracts alone, 12 were selected for full-text screening. Of the 12 full text studies, 6 were excluded due to language barrier (the title and abstract were in English, but the full text was in Chinese) (Melzack, 2001), having no visual artwork (Roseman, 2025), not having physical or emotional pain inducement (Melzack, 2001), and being review article (Melzack, 2001). 6 studies were included in the final analysis, and results are reported below.

### 3.2 Study outcomes

An overview of all study characteristics and outcomes can be found in Table 1. Studies were conducted in the United States (*n* = 3; 50%) (Richesin et al., 2021; Kaimal et al., 2019; Drake, 2019), China (*n* = 2; 33.33%) (Zhang et al., 2023; Yan et al., 2021), and Austria (*n* = 1; 16.67%) (Gebhart et al., 2020). Sample sizes varied widely across studies, from 34 to 66 participants, and participant ages in the included studies ranged from 17 to 72 years. While our initial eligibility criteria specified ages 18–60, two studies included participants slightly outside this range [17–49 (Gebhart et al., 2020) and 22–72 (Kaimal et al., 2019) years], and we retained them because they otherwise met all inclusion criteria and involved healthy adult populations. Participant sex varied across studies, with more females in all the studies.

**Table 1:** Overview of characteristics and outcomes of all included studies.

**Author (Year)**	**Population (n, gender, age)**	**Study design**	**Intervention (themes, materials, duration)**	**Emotional pain type & duration**	**Bio-marker**	**Pain/Emotion measure**	**Aim of study**	**Main results**	**Conclusion**
Zhang et al. (2023)	*n* = 44, F = 31, 19–29 yrs	Not specified	1) Emotional expression by drawing (venting task), oil paint sticks, 5 min 2) Distraction task by drawing a house, oil paint sticks, 5 min	Fear induction via film clip (1:54 min)	fNIRS	Affect Grid; subjective report; drawing coding methods	Compare venting vs distraction during drawing	Venting group showed less prefrontal activity (less cognitive control) and greater relaxation	Free emotional expression linked to higher positive valence
Richesin et al. (2021)	*n* = 44, F = 36, 18–24 yrs	RCT	1) Free drawing, color crayons and markers, 15 min 2) Free 3D drawing, VR (Google Tilt Brush), 15 min 3) Control, VR (HTC Vive office room), 15 min	Stress, anxiety, mood (pre/post interven-tion)	HR, skin conduc-tance, Salivary alpha-amylase (sAA)	PANAS; STAI; PSS	Compare 2D vs 3D art-making on stress, anxiety, mood	All groups improved mood/reduced anxiety; VR art group had greatest HR drop; sAA correlated with anxiety/ negative affect	Both 2D and 3D art-making reduce stress/anxiety; VR offers added benefits
Yan et al. (2021)	*n* = 59, F = 34, 18–27 yrs	Between-subjects	1) Free drawing, colored pencils, 510 s 2) Calculation task (continuous addition), colored pencils, 510 s	Emotion induction (sadness/ anger recall, 150 s)	fNIRS	Affect Grid (T1 rest, T2 emotion, T3 task)	Examine role of drawing in regulating sadness/ anger	Drawing led to minimal left DLPFC activation; effective in regulating sadness	Drawing regulates negative emotions, especially sadness
Gebhart et al. (2020)	*n* = 57, F = 44, 17–49 yrs	RCT	1) Animal-assisted therapy, dogs, 45–60 min 2) Music therapy, body percussion, 45–60 min 3) Mandala painting, different templates and colors, 45–60 min 4) Control, uninstructed, 60 min	Stress (pre/post interven-tion)	Salivary cortisol	VASS; STAI-State	Effects of distraction-focused interven-tions on exam stress/ anxiety	Interventions reduced stress/anxiety in daily life; biomarkers decreased significantly; no significant exam effect	Distraction has situation-dependent benefits
Kaimal et al. (2019)	*n* = 34, F = 27, 22–72 yrs	Mixed-methods	1) Open-studio art therapy instructed by art therapists, various art materials, 45 min 2) Active control coloring instructed by art therapists, pencils and markers, 45 min	Stress, self-efficacy, anxiety, burnout (pre/post)	Salivary cortisol, IL-6, CRP	PANAS; PSS; GSE; PROMIS; Maslach Burnout Inventory	Compare outcomes of coloring vs open-studio for caregivers of cancer patients	Both improved emotions, self-efficacy, reduced stress/anxiety/ burnout; coloring focus, open-studio support/ expression	Even brief art-making helps caregivers; repeated sessions benefits
Drake et al. (2019)	*n* = 66, F = 37, 18–36 yrs	RCT	1) Emotional expression by drawing, colored pencils, every week over a month, 10 min 2) Distraction task, colored pencils, every week over a month, 10min 3) Control, do nothing and just return to the lab after a month	Sad mood induction via visual imagery task (3-min)	HR, respirat-ory sinus arrhyth-mia (RSA)	PANAS, The Satisfaction With Life Scale	Compare expression vs distraction during drawing on mood	Drawing to distract improved mood more than drawing to express	psychological benefits of drawing can be both immediate and overtime, but psychophysiological benefits occur only over tme

### 3.3 Pain types

Populations included healthy people with no acute pain, while evaluating their emotional pain including: stress (*n* = 3; 50%) (Richesin et al., 2021; Kaimal et al., 2019; Gebhart et al., 2020), anxiety (*n* = 2; 33.33%) (Richesin et al., 2021; Kaimal et al., 2019), sadness (*n* = 2; 33.33%) (Drake, 2019; Yan et al., 2021), fear (*n* = 1; 16.67%) (Zhang et al., 2023), mood (*n* = 1; 16.67%) (Richesin et al., 2021), anger (*n* = 1; 16.67%) (Yan et al., 2021), self-efficacy and burnout (*n* = 1; 16.67%) (Kaimal et al., 2019). Moreover, Pain was evaluated in two ways in the included studies: by experimentally inducing emotional pain (fear, sadness, or anger) (*n* = 3; 50%)(Drake, 2019; Zhang et al., 2023; Yan et al., 2021) and by measuring emotional pain pre- and post-intervention (stress, anxiety, mood, self-efficacy, and burn out) (*n* = 3; 50%) (Richesin et al., 2021; Kaimal et al., 2019; Gebhart et al., 2020).

### 3.4 Types of visual art therapy

**Figure 3:**
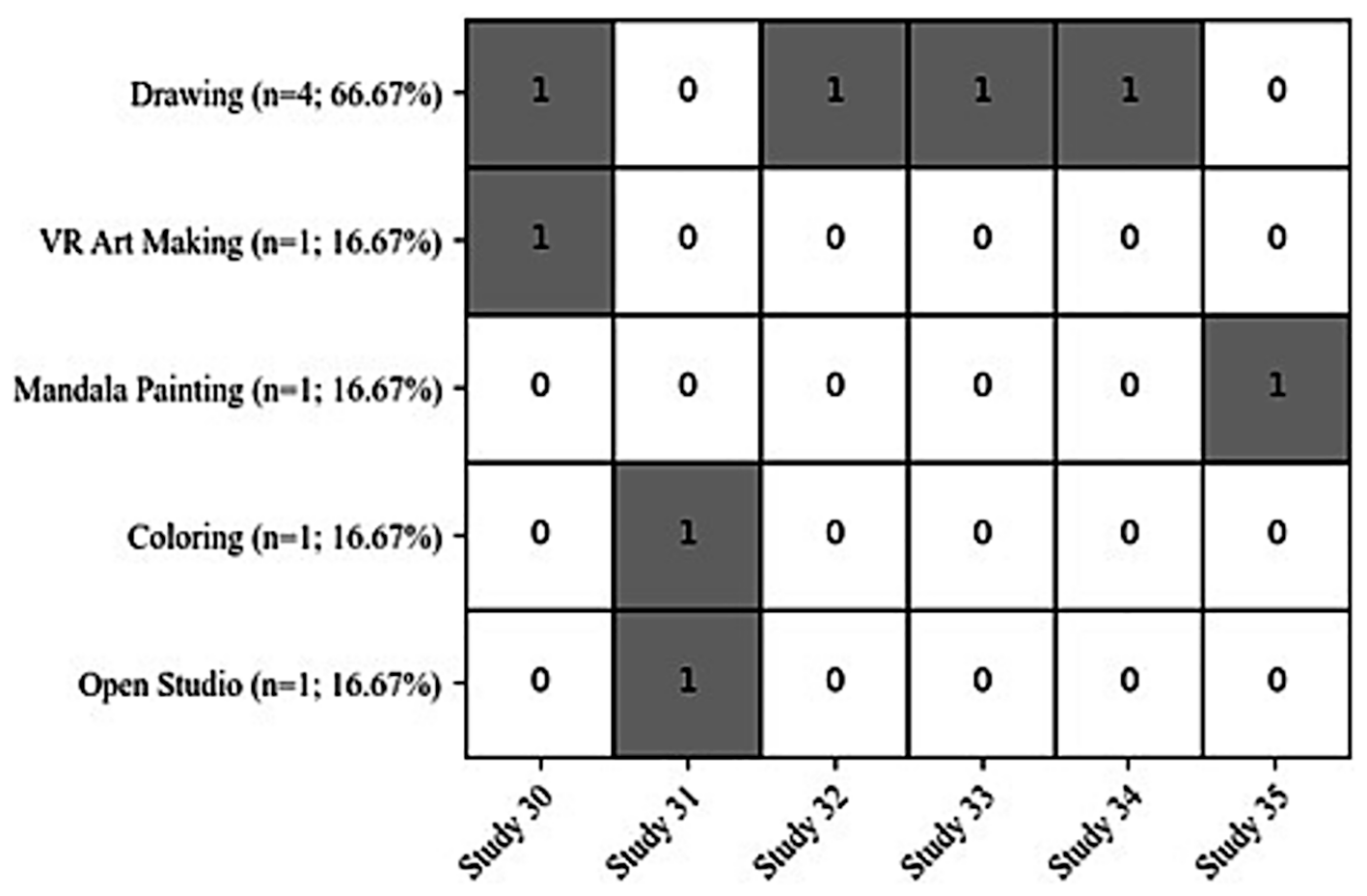
The distribution of visual art therapy types among the included studies (1 if the intervention was included in that study, or 0 if it was not).

The studies used following art therapy interventions including: drawing (*n* = 4; 66.67%) (Richesin et al., 2021; Drake, 2019; Zhang et al., 2023; Yan et al., 2021), art making in Virtual Reality (VR) (*n* = 1; 16.67%) (Richesin et al., 2021), mandala painting (*n* = 1; 16.67%) (Gebhart et al., 2020), coloring (*n* = 1; 16.67%) (Kaimal et al., 2019), open studio art therapy (*n* = 1; 16.67%) (Kaimal et al., 2019). Art therapy instructions were given (*n* = 4; 66.67%) (Richesin et al., 2021; Drake, 2019; Zhang et al., 2023; Yan et al., 2021) and in the rest of studies art therapy was run by professional and trained art therapist (*n* = 2; 33.33%) (Kaimal et al., 2019; Gebhart et al., 2020). For the art therapy intervention, tasks were chosen according to their therapeutic purpose including: free (non-directive) (*n* = 3; 50%) (Richesin et al., 2021; Kaimal et al., 2019; Yan et al., 2021), emotional expression (*n* = 2; 33.33%) (Drake, 2019; Zhang et al., 2023), distraction tasks (drawing a house or drawing from observation) (*n* = 2; 33.33%) (Drake, 2019; Zhang et al., 2023), mandala painting (*n* = 1; 16.67%) (Gebhart et al., 2020), and a coloring activity (*n* = 1; 16.67) (Kaimal et al., 2019). Figure 3 shows the distribution of different visual art therapies.

### 3.5 Duration of art therapy

In 5 studies, art therapy interventions were conducted in one session, but the duration of it varied to 5 min (*n* = 1; 16.67%) (Zhang et al., 2023), 8 min (*n* = 1; 16.67%) (Yan et al., 2021), 15 min (*n* = 1; 16.67%) (Richesin et al., 2021), 45 min (*n* = 1; 16.67%) (Kaimal et al., 2019), 45–60 min (*n* = 1; 16.67%) (Gebhart et al., 2020), and only one study reported four sessions of art therapy, each lasting 10 min (*n* = 1; 16.67%) (Drake, 2019). Study designs included randomized controlled trials (*n* = 3, 50%) (Richesin et al., 2021; Drake, 2019; Gebhart et al., 2020), between subject (*n* = 1, 16.67%) (Yan et al., 2021), mixed-methods (*n* = 1, 16.67%) (Kaimal et al., 2019), and not specified (*n* = 1; 16.67%).

### 3.6 Physiological and biological markers

**Figure 4:**
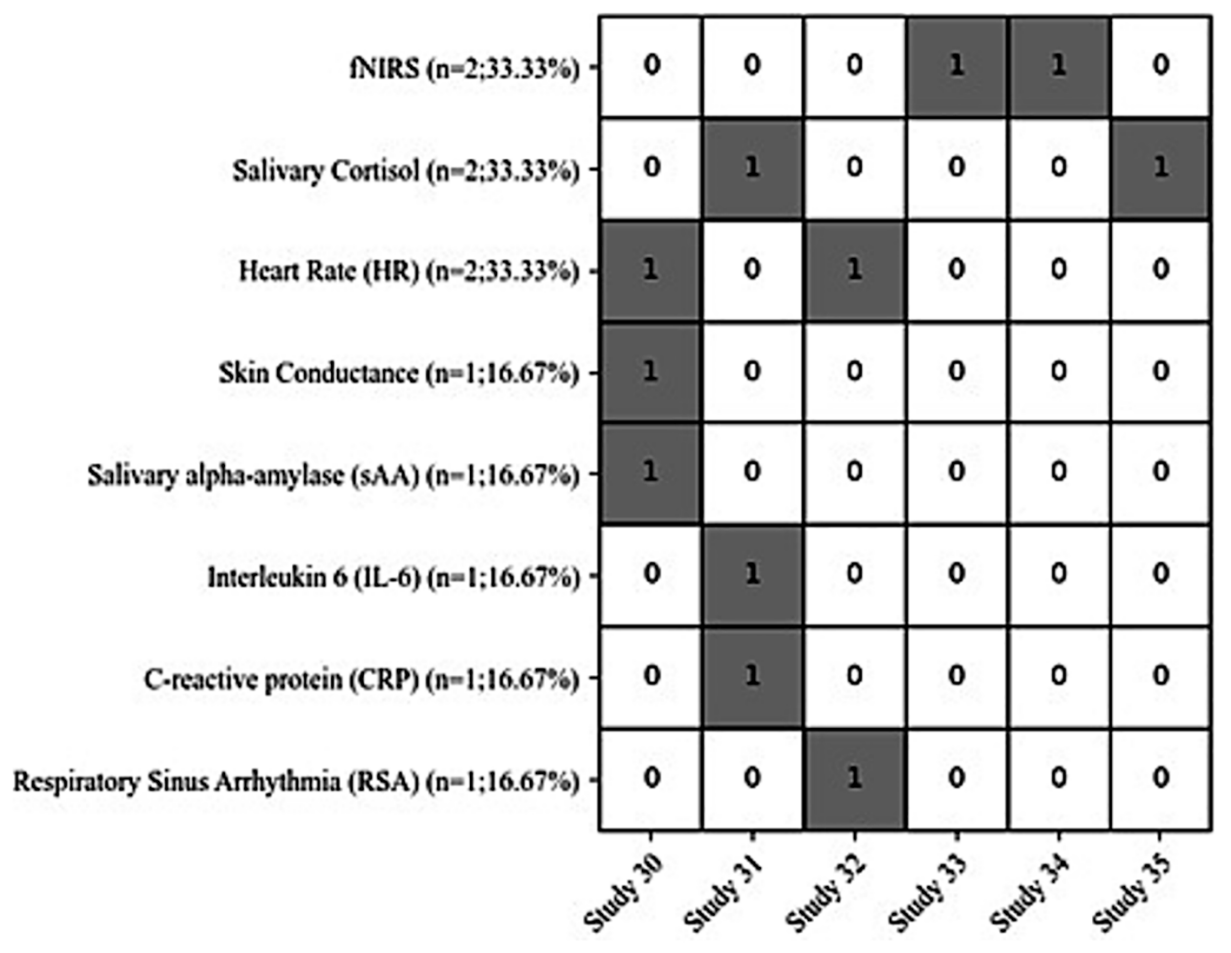
The distribution of physiological and biobased markers (1 if the marker was included in that study, or 0 if it was not).

Different physiological and biobased markers were measured to investigated the effect of art therapy, including: fNIRS (*n* = 2; 33.33%) (Zhang et al., 2023; Yan et al., 2021), Salivary Cortisol (*n* = 2; 33.33%) (Kaimal et al., 2019; Gebhart et al., 2020), Heart Rate (HR) (*n* = 2; 33.33%) (Richesin et al., 2021; Drake, 2019), skin conductance (*n* = 1; 16.67%) (Richesin et al., 2021), salivary alpha-amylase (sAA) (*n* = 1; 16.67%) (Richesin et al., 2021), interleukin 6 (IL-6) (*n* = 1; 16.67%) (Kaimal et al., 2019), C-reactive protein (CRP) (*n* = 1; 16.67%)(Kaimal et al., 2019), and respiratory sinus arrhythmia (RSA) (*n* = 1;16.67%) (Drake, 2019). Figure 4 shows the distribution of physiological and biobased markers in visual art therapy studies on pain.

### 3.7 Study findings

Findings from the studies suggest that visual art therapy can be helpful in improving mood and reducing stress, anxiety, fear, and sadness. Studies on fNIRS presented that art therapy resulted in minimal activation of the left dorsolateral prefrontal cortex (Zhang et al., 2023; Yan et al., 2021). Other studies noted the positive effect of art therapy in reducing stress, anxiety, and sad mood based on HR, Skin Conductance, Salivary Cortisol, sAA, IL-6, CRP, and RSA (Richesin et al., 2021; Kaimal et al., 2019; Drake, 2019; Gebhart et al., 2020). Some key recommendations that emerged from the studies were: small sample size, lack of control group, insufficient measurements.

### 3.8 Studies not fully meeting inclusion criteria

During the screening phase, some studies appeared interesting but did not fully meet the inclusion criteria. It was therefore decided to create two additional tables summarizing physiological and biological measurements: one focusing on visual art therapy without pain induction, and the other focusing on pain without visual art therapy.

#### 3.8.1 Physiological and biological measurements with visual art therapy

An overview of the characteristics and outcomes of these studies (*n* = 3) is provided in Table 2. Studies were conducted in Israel (*n* = 2; 66.67%) (Sion, 2019; Haiblum-Itskovitch et al., 2018), and Korea (*n* = 1; 33.33%) (Han D. H. et al., 2024). Sample sizes varied widely across studies, from 26 to 50 participants, and participant ages ranged from 19 to 55 years old. Participant sex varied across studies.

**Table 2:** Overview of studies including art therapy and biological measurements (no pain).

**Author (Year)**	**Population (n, gender, age)**	**Study design**	**Intervention (type, how, duration)**	**Biomarker**	**Emotion measure**	**Aim of study**	**Main results**	**Conclusion**
Han et al. (2024)	*n* = 26, F = 16, 20–29 yrs	Not specified	Consisted of 2 phases, first: four different art tasks with different materials (clay, marker pens, pencils, and colored pencils) each art task in 5 min, second phase drawing paper and tablet 5 min each	**f**NIRS	None	Investigating the relationship between art creation and brain function	Unique activation pattern in various brain regions based on the art activity	Barain is more activated during paper task than tablet-based task
Sion (2019)	*n* = 48, F = 48, 19–55 yrs	Between subjects	Digital art making, vs traditional art making with Oil pastels, free drawing, 45 min	HRV, RSA, CSI, Salivary cortisol	State-Trait Personality Inventory (STPI), SAM, Art-based intervention (ABI), FSS, Formal elements art therapy scale (FEATS)	Comparing digital vs traditional drawing	Both mediums were successful in regulating emotions	The traditional medium may create a more connected art-making experience
Haiblum-Itskovitch et al. (2018)	*n* = 50, F = 24, 22–44 yrs	Order rando-mizes	Drawing with pencil, oil-pastels, and gouache paint (10 min per each task)	HRV	Self-Report, SAM	Comparing the emotional and physiological responses to different art materials in fluidity	Drawing with gouache paint and oil-pastels improves positive mood.	Fluidity of the material is not the reason for the effectiveness of artwork. Oil-pastels results in unique emotional and physiological responses

##### 3.8.1.1 Types of visual art therapy

Different art materials and tasks were used across the studies, including: drawing with oil-pastels (*n* = 2; 66.67%) (Sion, 2019; Haiblum-Itskovitch et al., 2018), drawing with pencils (*n* = 2; 66.67%) (Haiblum-Itskovitch et al., 2018; Han D. H. et al., 2024), digital art making (*n* = 2; 66.67%) (Sion, 2019; Han D. H. et al., 2024), art making with clay, marker pens, and colored pencils (*n* = 1, 33.33%) (Han D. H. et al., 2024), drawing with gouache paint (*n* = 1; 33.33%) (Haiblum-Itskovitch et al., 2018). Art therapy instructions were given in all of them.

##### 3.8.1.2 Duration of art therapy

In all studies, art therapy interventions were conducted in one session, but the duration of it varied to 5 min (*n* = 1; 33.33%) (Han D. H. et al., 2024), 10 min (*n* = 1; 33.33%) (Haiblum-Itskovitch et al., 2018), and 45 min (*n* = 1; 33.33%) (Sion, 2019). Study designs included in between subject (*n* = 1; 33.33%) (Sion, 2019), order randomized (*n* = 1; 33.33%) (Haiblum-Itskovitch et al., 2018), and not specified (*n* = 1; 33.33%) (Han D. H. et al., 2024).

##### 3.8.1.3 Physiological and biological markers

Different physiological and biobased markers were measured to investigated the effect of art therapy, including: HRV (*n* = 2; 66.67%) (Sion, 2019; Haiblum-Itskovitch et al., 2018), fNIRS (*n* = 1; 33.33%) (Han D. H. et al., 2024), and RSA, Cardiac sympathetic index (CSI) (*n* = 1; 33.33%) (Sion, 2019).

##### 3.8.1.4 Study findings

Findings from the studies show that traditional art making is more effective than digital forms (Sion, 2019; Han D. H. et al., 2024). Moreover, the fluidity of traditional art materials is not an essential factor for their effectiveness (Haiblum-Itskovitch et al., 2018). Some key recommendations that emerged from the studies were: small sample size and single gender.

#### 3.8.2 Physiological and biological measurements with pain

Table 3 summarizes the characteristics and outcomes of the six studies identified. Studies were conducted in USA (*n* = 3; 50%) (Nichols et al., 2024; Weber et al., 2019; Groer et al., 2010), Brazil (*n* = 1; 16.67%) (Bortoletto et al., 2025), Germany (*n* = 1; 16.67%) (Weber et al., 2019), Netherlands (*n* = 1; 16.67%) (Bibbey et al., 2013), and UK (*n* = 1; 16.67%) (Steptoe et al., 2001). Sample sizes varied widely across studies, from 15 to 352 participants, and participant ages ranged from 19 to 64 years old. Participant sex varied across studies, except in one study where all participants were female (Nichols et al., 2024).

**Table 3:** Overview of studies including pain and biological measurements (no art therapy).

**Author (Year)**	**Population (n, gender, age)**	**Study design**	**Pain/emotion type & duration**	**Biomarker**	**Emotion measure**	**Aim of study**	**Main results**	**Conclusion**
Bortoletto et al. (2025)	*n* = 37, F = 13, 19–33 yrs	Randomized study	Stress-oriented (30 to 50 Seconds)	fMRI, Cardiovascular activity, blood CO_2_ level	None	Finding a paradigm to assess the body’s reactivity to stress	Stress elicited bilateral activation in the frontier frontal gyri, and altering the cardiovascular and blood CO_2_ level	Proposed protocol induces stress which is shown in fNIRS signals
Nichols et al. (2024)	*n* = 15, F = 15, 22–25 yrs	Not specified	Pressure-based pain by using standard blood pressure cuff (10 second)	fMRS	Generalized Anxiety Disorder-7 (GAD-7), Patient Health Questionnaire-9 (PHQ-9), Prodromal Questionnaire–Brief Version (PQ-B), Alcohol Dependence Scale (ADS), Nicotine Dependence Scale for Adolescents (NDSS-A), Severity of Dependence Scale (SDS), Graded Chronic Pain Scale (GCPS), Neuropathic Pain Scale (NPS).	Investigating the neurometabolite level in the dorsal anterior cingulate cortex (dASS) and Primary Somatosensory Cortex (SI) during acute pain	Increasing in glutamate levels following acute pain	Meaningful changes in (dASS) gamma aminobutyric acid in response to pain stimulation
Weber et al. (2019)	*n* = 33, F = 16, 23–44 yrs	Not specified	30 days of Isolation	EEG, Cortisol, Neurotrophic factors	PANAS-X, Cognitive test battery	Investigating the effect of short-term isolation on physiological and psychological parameters	Stress level increased, No further significant changes	30 days of isolation do not have a significant effect on brain activity and mood, just as the stress levels increased
Bibbey et al. (2013)	*n* = 352, F = 190, 57–60 yrs	Not specified	Acute psychological stress protocol comprising: a Stroop task, mirror tracking, and a speech task (5 min each)	Cardiovascular activity, Salivary cortisol	Using Big Five Inventory to assess neuroticism	Investigating the type of personality on stress reaction	Higher neuroticism scores, less agreeable and less open exhibited smaller cortisol and cardiovascular stress reactions	Negative personality disposition would be linked to diminished stress reactivity.
Groer et al. (2010)	*n* = 141, F = 27, 22–64 yrs	Randomized study	2 virtual reality scenarios for inducing stress: 1- motorcycle scenario (2 min) 2- workplace scenario (6 min)	Respiratory rate, ECG, Skin temperature, HR, and HRV, eye tracking, Saliva (Alpha amylase, Cortisol, IL-6, SIgA)	Perception of how stressful the experience (a single item scored on a 1 to 5 Likert scale)	Investigating the effect of a critical incident lethal force scenario on salivary biomarkers	“workplace” scenario rises the level of cortisol significantly	Virtual reality can produce stress
Steptoe et al. (2001)	*n* = 20, F = 12, 25–51 yrs	Not specified	Mental stress including 2 computer based tasks (5 min each)	HR, Blood Pressure, Saliva, TNF-*a,* IL-6, and IL-1Ra	Behavioral performance of the tasks	investigate whether acute psychological stress leads to changes in circulating pro-inflammatory cytokines	significant increases in IL-6 and IL-1Ra concentrations in the stress group	inflammatory cytokines respond to stress increases cytokine responses associated sympathetic reactivity

##### 3.8.2.1 Types of pain

Populations included healthy people with no acute pain, while evaluating their pain, including: stress (*n* = 4; 66.67%) (Groer et al., 2010; Bortoletto et al., 2025; Bibbey et al., 2013; Steptoe et al., 2001), pressure-based pain (*n* = 1; 16.67%) (Nichols et al., 2024), isolation (*n* = 1; 16.67%) (Weber et al., 2019).

##### 3.8.2.2 Physiological and biological markers

Different physiological and biobased markers were measured to measure the pain, including: cardiovascular activities (*n* = 4; 66.67%) (Groer et al., 2010; Bortoletto et al., 2025; Bibbey et al., 2013; Steptoe et al., 2001), Saliva (*n* = 3; 50%) (Groer et al., 2010; Bibbey et al., 2013; Steptoe et al., 2001), fMRI (*n* = 1; 16.67%) (Bortoletto et al., 2025), Functional Magnetic Resonance Spectroscopy (fMRS) (*n* = 1; 16.67%) (Nichols et al., 2024), EEG (*n* = 1; 16.67%) (Weber et al., 2019), blood CO_2_ levels (*n* = 1; 16.67%) (Bortoletto et al., 2025) and Cortisol, Neurotrophic factors from blood (*n* = 1; 16.67%) (Weber et al., 2019).

##### 3.8.2.3 Study findings

The findings indicate that pain can modulate various physiological and biological parameters. fMRI study has shown the brain activation in the frontier frontal gyri elicited by stress (Bortoletto et al., 2025). fMRS study has reported that acute pain is associated with increased glutamate levels(Nichols et al., 2024). In contrast, social isolation did not alter brain activity but was associated with increased stress levels (Weber et al., 2019). Additionally, negative personality traits can influence stress reactivity (Bibbey et al., 2013). Inducing stress in a virtual reality platform has been shown to effectively produce stress (Groer et al., 2010). The study shows that acute mental stress triggers delayed cytokine responses, and that these responses vary between individuals depending on sympathetic nervous system activity (Steptoe et al., 2001). Some key limitations and recommendations from these studies include small sample sizes and the inclusion of a single gender.

## 4 Discussion

This scoping review identified and evaluated the existing evidence on art therapy interventions for emotional pain in healthy populations with a focus on sensory and biological biomarkers. Six research papers were identified as eligible. The main findings from these studies indicate that art therapy can help reduce stress, anxiety, and negative emotions, as well as enhance positive mood. This claim is supported by physiological and biological biomarkers, alongside self-report questionnaires. In addition, the research suggests that art therapy alters the activation of different brain regions. Therefore, art therapy may contribute to improvements in emotional pain. However, to the best of our knowledge, studies that combine art therapy with experimentally induced physical pain and use sensory or neuroimaging parameters in healthy adults are missing. Future research addressing this gap could help clarify these effects.

While no previous review has directly examined art therapy’s effects on experimentally induced physical pain in healthy adults, findings from clinical and vulnerable populations may offer valuable insights and serve as a model for future mechanistic research in non-clinical settings. A scoping review focusing on children reported that art therapy interventions can reduce pain, anxiety, stress, and fear associated with treatment (Olaizola et al., 2024), in addition a narrative review highlighted consistent improvements in pain, mood, stress, and quality of life among patients with chronic pain (Raudenská et al., 2023). Both findings align with the present scoping review, which shows reductions in stress, anxiety, and negative feelings in healthy adults. Similarly, another scoping review on chronic non-cancer pain found that despite variations in samples and art modalities, arts-based methods were considered suitable and highly effective for addressing chronic pain in patients (Harasymchuk et al., 2024). This resonates with our findings that, regardless of the materials used, art therapy can be helpful for relieving emotional pain. Evidence from another systematic review demonstrated that art-based interventions were effective for children undergoing venipuncture, reducing treatment-related distress (Suleman et al., 2023). Finally, a review on pediatric cancer care emphasized that integrating art therapy can yield positive physical and psychological outcomes in palliative care settings (Motlagh et al., 2023). Taken together, these reviews demonstrate the potential of art therapy for pain management across diverse populations; however, this scoping review extends this body of knowledge by identifying a gap in research on healthy individuals, where the effects of art therapy on induced physical pain remain largely unexplored.

This scoping review found no studies that investigated the effect of visual art therapy on physical pain in healthy populations using physiological or biological measures, Further research is needed to investigate its direct impact on physical pain and to better understand its mechanisms of action. All included studies focused on emotional pain. With regard to neuroimaging, existing research demonstrated brain activation during visual art therapy but relied exclusively on fNIRS, leaving a clear gap for EEG-based investigations (Zhang et al., 2023; Yan et al., 2021). Furthermore, all included studies compared visual art therapy with other interventions or forms of art therapy. Future studies could explore the effects of different visual art materials and tasks to determine whether specific materials or tasks yield distinct therapeutic outcomes and what their therapeutic mechanisms are. This knowledge would aid building art therapy interventions and programs bridging psychological, physiological, and biological aspects of pain that are based on sound biopsychosocial evidence. Depending on the type of pain, it would also be beneficial to include objective measures of pain intensity, such as salivary biomarkers or heart rate (Richesin et al., 2021; Kaimal et al., 2019; Drake, 2019; Gebhart et al., 2020). Since personality may influence stress reactivity, incorporating questionnaires on personality traits and emotional background could provide additional insight into the effects of visual art therapy on pain (Bibbey et al., 2013). Another recurring limitation across studies was the use of small sample sizes. Most included studies are small and exploratory in nature, limiting statistical power and clinical generalizability. Therefore, the findings should be interpreted as preliminary and hypothesis-generating. Because this is a scoping review, clinical significance has not been assessed but instead mapped outcome measures and physiological markers used across studies. Future research should therefore prioritize larger, more diverse samples and employ a wider range of neuroimaging and biological measures (as an example, EEG, biomarkers). Such work may enable the identification of biomarkers for visual art therapy across different types of pain. Identifying such biomarkers could guide the development of tailored art therapy interventions, for example by determining which approaches are most effective for specific types of pain and appropriate durations of practice. Building this evidence base would also support the integration of visual art therapy into healthcare systems and strengthen arguments for its coverage by health insurance.

To better integrate the Supplementary material with the main findings, the overview tables are used to contextualize how existing studies operationalize art therapy-related interventions and physiological outcome measures. These tables highlight recurring methodological patterns, including the frequent use of short, single-session interventions, small sample sizes, and a limited range of biological and neurophysiological markers. By mapping these characteristics, the review helps identify which biomarkers (e.g., heart rate variability, cortisol, fNIRS) have been most commonly applied and where important gaps remain. This synthesis provides a foundation for informing future research design, including the selection of outcome measures, intervention duration, and multimodal assessment strategies in studies of art-based interventions. At a broader level, the structured overview of existing evidence may support program development and policy discussions by illustrating how physiological and biological outcomes have been operationalized to date, while also underscoring the need for more robust, standardized, and context-sensitive approaches before wider implementation can be recommended.

## 5 Conclusion

This scoping review mapped the current evidence on visual art therapy and emotional pain. Overall, the evidence indicates that art therapy may reduce stress, anxiety, and negative emotions while enhancing positive mood. These effects are supported by both self-report measures and physiological and biological biomarkers. Furthermore, neuroimaging studies suggest that art therapy influences activity in specific brain regions, pointing to its potential role in alleviating emotional pain. Existing studies primarily examined emotional pain, with no studies investigating its effects on physical pain in healthy populations using physiological or biological measures. Future research should prioritize larger, more diverse samples, expand the use of neuroimaging and biological measures, and examine how different forms of art therapy may affect various types of pain. Building this evidence base may ultimately enable the identification of biomarkers for visual art therapy, inform tailored interventions, and support its integration into healthcare systems.

## Data Availability

The original contributions presented in the study are included in the article/Supplementary material, further inquiries can be directed to the corresponding author.

## Footnotes

1
https://www.rayyan.ai

## Supplementary material

APPENDIX A Example of database search strategy (MEDLINE).

## References

[ref1] Aaron R. V. Ravyts S. G. Carnahan N. D. Bhattiprolu K. Harte N. McCaulley C. C. . (2025). Prevalence of depression and anxiety among adults with chronic pain: a systematic review and meta-analysis. JAMA Netw. Open 8:e250268. doi: 10.1001/jamanetworkopen.2025.0268 40053352 PMC11889470

[ref2] Arksey H. O'malley L. (2005). Scoping studies: towards a methodological framework. Int. J. Soc. Res. Methodol. 8, 19–32. doi: 10.1080/1364557032000119616

[ref3] Barnett K. S. Vasiu F. (2024). How the arts heal: a review of the neural mechanisms behind the therapeutic effects of creative arts on mental and physical health. Front. Behav. Neurosci. 18:1422361. doi: 10.3389/fnbeh.2024.1422361 39416439 PMC11480958

[ref4] Beerse M. E. Van Lith T. Pickett S. M. Stanwood G. D. (2020). Biobehavioral utility of mindfulness-based art therapy: Neurobiological underpinnings and mental health impacts. Exp. Biol. Med. 245, 122–130. doi: 10.1177/1535370219883634, 31635490 PMC7016419

[ref5] Bibbey A. Carroll D. Roseboom T. J. Phillips A. C. de Rooij S. R. (2013). Personality and physiological reactions to acute psychological stress. Int. J. Psychophysiol. 90, 28–36. doi: 10.1016/j.ijpsycho.2012.10.018, 23147393

[ref6] Birtwistle S. Rule S. P. Wilson V. (2023). The Scottish health survey: main report. 2022nd Edn. Edinburgh: Scottish Government.

[ref7] Bortoletto L. F. Martins G. Yamamoto B. de Lima B. Sanchez V. Mesquita R. (2025). Characterizing neural and systemic responses during acute psychosocial stress using functional near infrared spectroscopy. Bellingham, WA, USA: SPIE.

[ref8] Breivik H. Collett B. Ventafridda V. Cohen R. Gallacher D. (2006). Survey of chronic pain in Europe: prevalence, impact on daily life, and treatment. Eur. J. Pain 10, 287–333. doi: 10.1016/j.ejpain.2005.06.009 16095934

[ref9] Bugos J. A. Bidelman G. M. Moreno S. Shen D. Lu J. Alain C. (2022). Music and visual art training increase auditory-evoked theta oscillations in older adults. Brain Sci. 12:1300. doi: 10.3390/brainsci12101300 36291234 PMC9599228

[ref10] Daly M. Macchia L. (2023). Global trends in emotional distress. Proc. Natl. Acad. Sci. 120:e2216207120. doi: 10.1073/pnas.2216207120 36972447 PMC10083620

[ref12] Drake J. E. (2019). Examining the psychological and psychophysiological benefits of drawing over one month. Psychol. Aesthet. Creat. Arts 13, 338–347. doi: 10.1037/aca0000179

[ref13] Dueñas M. Ojeda B. Salazar A. Mico J. A. Failde I. (2016). A review of chronic pain impact on patients, their social environment and the health care system. J. Pain Res. 9, 457–467. doi: 10.2147/JPR.S105892, 27418853 PMC4935027

[ref14] Estancial Fernandes C. S. Lima M. G. Barros M. B. A. (2019). Emotional problems and health-related quality of life: population-based study. Qual. Life Res. 28, 3037–3046. doi: 10.1007/s11136-019-02230-9, 31240538

[ref15] Gebhart V. Buchberger W. Klotz I. Neururer S. Rungg C. Tucek G. . (2020). Distraction-focused interventions on examination stress in nursing students: effects on psychological stress and biomarker levels. A randomized controlled trial. Int. J. Nurs. Pract. 26:e12788. doi: 10.1111/ijn.12788, 31724291

[ref16] Gierthmühlen J. Enax-Krumova E. K. Attal N. Bouhassira D. Cruccu G. Finnerup N. B. . (2015). Who is healthy? Aspects to consider when including healthy volunteers in QST--based studies-a consensus statement by the EUROPAIN and NEUROPAIN consortia. Pain 156, 2203–2211. doi: 10.1097/j.pain.0000000000000227, 26075963

[ref17] Godinho F. Magnin M. Frot M. Perchet C. Garcia-Larrea L. (2006). Emotional modulation of pain: is it the sensation or what we recall? J. Neurosci. 26, 11454–11461. doi: 10.1523/JNEUROSCI.2260-06.2006, 17079675 PMC6674534

[ref18] Groer M. Murphy R. Bunnell W. Salomon K. Van Eepoel J. Rankin B. . (2010). Salivary measures of stress and immunity in police officers engaged in simulated critical incident scenarios. J. Occup. Environ. Med. 52, 595–602. doi: 10.1097/JOM.0b013e3181e129da, 20523239

[ref19] Gruber H. Oepen R. (2018). Emotion regulation strategies and effects in art-making: a narrative synthesis. Arts Psychother. 59, 65–74. doi: 10.1016/j.aip.2017.12.006

[ref20] Gussak D. E. Rosal M. L. (2026). “Introduction” in eds. D. E. Gussak and M. L. Rosal. The wiley handbook of art therapy, Chichester, UK: John Wiley & Sons. 1–4.

[ref21] Haiblum-Itskovitch S. Czamanski-Cohen J. Galili G. (2018). Emotional response and changes in heart rate variability following art-making with three different art materials. Front. Psychol. 9:968. doi: 10.3389/fpsyg.2018.00968 29967587 PMC6015920

[ref22] Hallegraeff J. M. Kan R. van Trijffel E. Reneman M. F. (2020). State anxiety improves prediction of pain and pain-related disability after 12 weeks in patients with acute low back pain: a cohort study. J. Physiother. 66, 39–44. doi: 10.1016/j.jphys.2019.11.011, 31862258

[ref23] Han B. Jia Y. Hu G. Bai L. Gains H. You S. . (2024). The effects of visual art therapy on adults with depressive symptoms: a systematic review and meta-analysis. Int. J. Ment. Health Nurs. 33, 1183–1196. doi: 10.1111/inm.13331, 38606659

[ref24] Han D. H. Kim S. K. Kim S. (2024). Brain activation in response to art-based tasks using diverse materials based on the Expressive Therapy Continuum (ETC). Arts Psychother. 90:102185. doi: 10.1016/j.aip.2024.102185

[ref25] Harasymchuk S. J. Howard A. F. Noga H. Kelly M. T. Yong P. J. (2024). The use of arts-based research in chronic pain: a scoping review. Can J Pain. 8:2352876. doi: 10.1080/24740527.2024.2352876, 38915305 PMC11195486

[ref26] Hawker G. A. Mian S. Kendzerska T. French M. (2011). Measures of adult pain: visual analog scale for pain (VAS Pain), numeric rating scale for pain (NRS Pain), McGill pain questionnaire (MPQ), short-form Mcgill pain questionnaire (SF-MPQ), chronic pain grade scale (CPGS), short form-36 bodily pain scale (SF-36 BPS), and measure of intermittent and constant osteoarthritis pain (ICOAP). Arthritis Care Res. 63, S240–S252. doi: 10.1002/acr.20543 22588748

[ref27] Hill A. K. G. (1945). Art versus illness, a story of art therapy. London: G. Allen and Unwin.

[ref11] Hills de Zárate M Waller D. Vaculik C. L. (2025). The Routledge international handbook of art therapy practice. New York, NY, USA: Routledge.

[ref28] Hohls J. K. König H. H. Quirke E. Hajek A. (2021). Anxiety, depression and quality of life-a systematic review of evidence from longitudinal observational studies. Int. J. Environ. Res. Public Health 18:12022. doi: 10.3390/ijerph182212022 34831779 PMC8621394

[ref29] Joschko R. Klatte C. Grabowska W. A. Roll S. Berghöfer A. Willich S. N. (2024). Active visual art therapy and health outcomes: a systematic review and meta-analysis. JAMA Netw. Open 7:e2428709. doi: 10.1001/jamanetworkopen.2024.28709 39264631 PMC11393726

[ref30] Kaimal G. Ayaz H. Herres J. Dieterich-Hartwell R. Makwana B. Kaiser D. H. . (2017). Functional near-infrared spectroscopy assessment of reward perception based on visual self-expression: coloring, doodling, and free drawing. Arts Psychother. 55, 85–92. doi: 10.1016/j.aip.2017.05.004

[ref31] Kaimal G. Carroll-Haskins K. Mensinger J. L. Dieterich-Hartwell R. M. Manders E. Levin W. P. (2019). Outcomes of art therapy and coloring for professional and informal caregivers of patients in a radiation oncology unit: a mixed methods pilot study. Eur. J. Oncol. Nurs. 42, 153–161. doi: 10.1016/j.ejon.2019.08.006, 31557665

[ref32] Kaimal G. Ray K. Muniz J. (2016). Reduction of cortisol levels and participants' responses following art making. Art Ther. 33, 74–80. doi: 10.1080/07421656.2016.1166832, 27695158 PMC5004743

[ref33] Kim K. S. Lor M. Rakel B. (2023). Evaluation of art making activity as a pain management strategy for older adults and their experience using an art making intervention. Geriatr. Nurs. 50, 109–116. doi: 10.1016/j.gerinurse.2023.01.007, 36774677

[ref34] Kret M. E. Ploeger A. (2015). Emotion processing deficits: a liability spectrum providing insight into comorbidity of mental disorders. Neurosci. Biobehav. Rev. 52, 153–171. doi: 10.1016/j.neubiorev.2015.02.011, 25725415

[ref35] Levac D. Colquhoun H. O'Brien K. K. (2010). Scoping studies: advancing the methodology. Implement. Sci. 5:69. doi: 10.1186/1748-5908-5-69, 20854677 PMC2954944

[ref36] Lim J.-w. Zebrack B. (2004). Caring for family members with chronic physical illness: a critical review of caregiver literature. Health Qual. Life Outcomes 2:50. doi: 10.1186/1477-7525-2-50 15377384 PMC521496

[ref37] Lockwood C. Dos Santos K. B. Pap R. (2019). Practical guidance for knowledge synthesis: scoping review methods. Asian Nurs. Res. 13, 287–294. doi: 10.1016/j.anr.2019.11.002, 31756513

[ref38] Lumley M. A. Cohen J. L. Borszcz G. S. Cano A. Radcliffe A. M. Porter L. S. . (2011). Pain and emotion: a biopsychosocial review of recent research. J. Clin. Psychol. 67, 942–968. doi: 10.1002/jclp.20816, 21647882 PMC3152687

[ref39] Melzack R. (2001). Pain and the neuromatrix in the brain. J. Dent. Educ. 65, 1378–1382. doi: 10.1002/j.0022-0337.2001.65.12.tb03497.x 11780656

[ref40] Motlagh E. G. Bakhshi M. Davoudi N. Ghasemi A. Moonaghi H. K. (2023). The physical and psychological outcomes of art therapy in pediatric palliative care: a systematic review. J. Res. Med. Sci. 28:13. doi: 10.4103/jrms.jrms_268_22 37064791 PMC10098136

[ref41] Nan J. K. M. Hinz L. D. Lusebrink V. B. (2021). “Chapter 42—clay art therapy on emotion regulation: research, theoretical underpinnings, and treatment mechanisms” in The neuroscience of depression. eds. Martin C. R. Hunter L.-A. Patel V. B. Preedy V. R. Rajendram R. (Amsterdam, the Netherlands: Academic Press), 431–442.

[ref42] Naumburg M. (1987). Dynamically oriented art therapy: its principles and practices: illustrated with three case studies. New York City: Magnolia Street Publishers.

[ref43] Nichols S. J. Yanes J. A. Reid M. A. Robinson J. L. (2024). 7 T characterization of excitatory and inhibitory systems of acute pain in healthy female participants. NMR Biomed. 37:e5088. doi: 10.1002/nbm.5088, 38140895

[ref44] Olaizola S. Lalloo C. Vickers V. Kelenc L. Tariq S. Brown S. C. . (2024). Art therapy for paediatric pain: a scoping review. Children 11:619. doi: 10.3390/children11060619, 38929199 PMC11202121

[ref45] Ouzzani M. Hammady H. Fedorowicz Z. Elmagarmid A. (2016). Rayyan—a web and mobile app for systematic reviews. Syst. Rev. 5:210. doi: 10.1186/s13643-016-0384-4, 27919275 PMC5139140

[ref46] Pandelani F. F. Nyalunga S. L. N. Mogotsi M. M. Mkhatshwa V. B. (2023). Chronic pain: its impact on the quality of life and gender. Front. Pain Res. 4:1253460. doi: 10.3389/fpain.2023.1253460 PMC10534032 37781217

[ref47] Piao X. Xie J. Managi S. (2024). Continuous worsening of population emotional stress globally: universality and variations. BMC Public Health 24:3576. doi: 10.1186/s12889-024-20961-4, 39716139 PMC11668040

[ref48] Raudenská J. Šteinerová V. Vodičková Š. Raudenský M. Fulková M. Urits I. . (2023). Arts therapy and its implications in chronic Pain management: a narrative review. Pain Ther. 12, 1309–1337. doi: 10.1007/s40122-023-00542-w, 37733173 PMC10616022

[ref49] Raymond T. J. Tobin K. A. Rogers T. S. (2021). Nonopioid pharmacologic treatments for chronic pain. Am. Fam. Physician 103, 561–565. 33929169

[ref50] Richesin M. T. Baldwin D. R. Wicks L. A. M. (2021). Art making and virtual reality: a comparison study of physiological and psychological outcomes. Arts Psychother. 75:101823. doi: 10.1016/j.aip.2021.101823

[ref51] Roseman I. J. (2025). An emotion system theory to address gaps in affective intelligence theory and conceptualization of emotional phenomena. Front. Polit. Sci. 7:1570686.

[ref52] Shi Y. Wu W. (2023). Multimodal non-invasive non-pharmacological therapies for chronic pain: mechanisms and progress. BMC Med. 21:372. doi: 10.1186/s12916-023-03076-2, 37775758 PMC10542257

[ref53] Sion A. (2019). Is drawing with oil pastels more relaxing than drawing on a tablet? A comparison of autonomic nervous system measures, the undo function, experience reports and drawings of participants making art after stress. [Master's]. Haifa: University of Haifa (Israel).

[ref54] Slavich G. M. Way B. M. Eisenberger N. I. Taylor S. E. (2010). Neural sensitivity to social rejection is associated with inflammatory responses to social stress. Proc. Natl. Acad. Sci. USA 107, 14817–14822. doi: 10.1073/pnas.1009164107 20679216 PMC2930449

[ref55] Steptoe A. Willemsen G. Owen N. Flower L. Mohamed-Ali V. (2001). Acute mental stress elicits delayed increases in circulating inflammatory cytokine levels. Clin. Sci. 101, 185–192. doi: 10.1042/cs1010185, 11473494

[ref56] Stubhaug A. Hansen J. L. Hallberg S. Gustavsson A. Eggen A. E. Nielsen C. S. (2024). The costs of chronic pain-Long-term estimates. Eur. J. Pain 28, 960–977. doi: 10.1002/ejp.2234, 38214661

[ref57] Sturgeon J. A. Zautra A. J. (2016). Social pain and physical pain: shared paths to resilience. Pain Manag. 6, 63–74. doi: 10.2217/pmt.15.56, 26678402 PMC4869967

[ref58] Suleman S. Halek M. Enskär K. Atrushi A. Nilsson S. (2023). A systematic review and meta-analysis of the effect of art-based psychological distraction on school-aged children’s pain and anxiety during painful procedures. Pieleg. XXI Wieku 22, 264–272. doi: 10.2478/pielxxiw-2023-0031

[ref59] Tricco A. C. Lillie E. Zarin W. O'Brien K. K. Colquhoun H. Levac D. . (2018). PRISMA extension for scoping reviews (PRISMA-ScR): checklist and explanation. Ann. Intern. Med. 169, 467–473. doi: 10.7326/M18-0850, 30178033

[ref60] Weber J. Javelle F. Klein T. Foitschik T. Crucian B. Schneider S. . (2019). Neurophysiological, neuropsychological, and cognitive effects of 30 days of isolation. Exp. Brain Res. 237, 1563–1573. doi: 10.1007/s00221-019-05531-0, 30927043

[ref61] Yan W. Zhang M. Liu Y. (2021). Regulatory effect of drawing on negative emotion: a functional near-infrared spectroscopy study. Arts Psychother. 74:101780. doi: 10.1016/j.aip.2021.101780

[ref62] Zhang Z. Gewandter J. S. Geha P. (2021). Brain imaging biomarkers for chronic pain. Front. Neurol. 12:734821. doi: 10.3389/fneur.2021.734821, 35046881 PMC8763372

[ref63] Zhang X. Yan W. Xu C. Yang A. Shen Z. Guo X. (2023). Functional near-infrared spectroscopy approach to the emotional regulation effect of drawing: venting versus distraction. Brain Behav. 13:e3248. doi: 10.1002/brb3.3248, 37700566 PMC10636421

