# Supplementary material for: Art therapy and emotional pain: a scoping review of physiological and biological measures
Source: Front Hum Neurosci. 2026 Mar 11;20:1736930. doi: 10.3389/fnhum.2026.1736930 (PMC13013050; doi:10.3389/fnhum.2026.1736930)
Supplement: APPENDIX A — Example of database search strategy (MEDLINE). [file Supplementary_file_1.pdf]

Table 1. Overview of characteristics and outcomes of all included studies

Author (Year)	Country	Journal	DOI	Population (n, gender, age)	Study design	Intervention (themes, materials, duration)	Emotional pain type & duration	Physiological marker	Biomarker	Pain/Emotion measure	Aim of study	Main results	Conclusion	Limitations	Notes
**Zhang et al. (2023)**	China	Brain and Behavior	10.1002/brb3.3248	n=44, F=31, 19–29 yrs	Not specified	1) Emotional expression by drawing (venting task), oil paint sticks, 5 min 2) Distraction task by drawing a house, oil paint sticks, 5 min	Fear induction via film clip (1:54 min)	fNIRS	None	Affect Grid; subjective report; drawing coding methods	Compare venting vs distraction during drawing	Venting group showed less prefrontal activity (less cognitive control) and greater relaxation	Free emotional expression linked to higher positive valence	No additional physiological measures (e.g., skin conductance)	Fear induced by showing film Dead Silence
**Richesin et al. (2021)**	USA	The Arts in Psychotherapy	10.1016/j.aip.2021.101823	n=44, F=36, 18–24 yrs	RCT	1) Free drawing, color crayons and markers, 15 min 2) Free 3D drawing, VR (Google Tilt Brush), 15 min 3) Control, VR (HTC Vive office room), 15 min	Stress, anxiety, mood (pre/post intervention)	HR, skin conductance	Salivary alpha-amylase (sAA)	PANAS; STAI; PSS	Compare 2D vs 3D art-making on stress, anxiety, mood	All groups improved mood/reduced anxiety; VR art group had greatest HR drop; sAA correlated with anxiety/negative affect	Both 2D and 3D art-making reduce stress/anxiety; VR offers added benefits	Small sample; omission of residual change in analysis	Randomized controlled trial
**Yan et al. (2021)**	China	The Arts in Psychotherapy	10.1016/j.aip.2021.101780	n=59, F=34, 18–27 yrs	Between-subjects	1) Free drawing, colored pencils, 510 s 2) Calculation task (continuous addition), colored pencils, 510 s	Emotion induction (sadness/anger recall, 150 s)	fNIRS	None	Affect Grid (T1 rest, T2 emotion, T3 task)	Examine role of drawing in regulating sadness/anger	Drawing led to minimal left DLPFC activation; effective in regulating sadness	Drawing regulates negative emotions, especially sadness	Focus only on prefrontal activity; only free drawing tested	Participants randomized to anger/sadness × drawing/calculation
**Gebhart et al. (2020)**	Austria	Int. J. Nurs. Pract.	10.1111/ijn.12788	n=57, F=44, 17–49 yrs	RCT	1) Animal-assisted therapy, dogs, 45–60 min 2) Music therapy, body percussion, 45–60 min 3) Mandala painting, different templates and	Stress (pre/post intervention)	None	Salivary cortisol	VASS; STAI-State	Effects of distraction-focused interventions on exam stress/anxiety	Interventions reduced stress/anxiety in daily life; biomarkers decreased significantly; no significant exam effect	Distraction has situation-dependent benefits	Small sample per group	----

						colors, 45–60 min<br>4) Control, uninstructed free hour, 60 min									
**Kaimal et al. (2019)**	USA	Eur. J. Oncol. Nurs.	10.1016/j.ejon.Done2019.08.006	n=34, F=27, 22–72 yrs	Mixed-methods	1) Open-studio art therapy instructed by art therapists, various art materials, 45 min<br>2) Active control coloring instructed by art therapists, pencils and markers, 45 min	Stress, self-efficacy, anxiety, burnout (pre/post)	None	Salivary cortisol, IL-6, CRP	PANAS; PSS; GSE; PROMIS; Maslach Burnout Inventory	Compare outcomes of coloring vs open-studio for caregivers of cancer patients	Both improved emotions, self-efficacy, reduced stress/anxiety/burnout; coloring focus, open-studio support/expression	Even brief art-making helps caregivers; repeated sessions benefits	Small sample; no control group; biomarker collection incomplete	Caregivers of radiation oncology patients
**Jennifer E. Drake et al (2019)**	USA	Psychology of Aesthetics, Creativity, and the Arts	https://doi.org/10.1037/aca0000179	n=66, F= 37, 18-36 yrs	RCT	1) Emotional expression by drawing, colored pencils, every week over a month, 4 times in total each time 10 min<br>2) Distraction task, colored pencils, every week over a month, 4 times in total each time 10<br>3) Control, do nothing and just return to the lab after a month	Sad mood induction via visual imagery task (3-min)	HR, respiratory sinus arrhythmia (RSA)	None	PANAS, The Satisfaction With Life Scale	Compare expression vs distraction during drawing on mood	Drawing to distract improved mood more than drawing to express	psychological benefits of drawing can be both immediate and overtime, but psychophysiological benefits occur only over tme	Control condition did not engaged in any activity	In drawing tasks, they should draw in respective condition for 10 min each week over a month

Table 2-Overview of studies including art therapy and biological measurements (no pain)

**Author (Year)**	**Country**	**Journal**	**DOI**	**Population (n, gender, age)**	**Study design**	**Intervention (type, how, duration)**	**Physiological marker**	**Biomarker**	**Emotion measure**	**Aim of study**	**Main results**	**Conclusion**	**Limitations**	**Notes**
**Doug Hyun Hanet al (2024)**	Korea	The Arts in Psychotherapy	https://doi.org/10.1016/j.aip.2024.102185	n=26, F=16, 20-29yrs	Not specified	Consisted of 2 phases, first: four different art tasks with different materials (clay, marker pens, pencils, and colored pencils) each art task in 5 min, second phase drawing paper and tablet 5 min each	fNIRS	None	None	Investigating the relationship between art creation and brain function	Unique activation pattern in various brain regions based on the art activity	Barain is more activated during paper task than tablet-based task	Small sample size	--
**Aviv Sion (2019)**	Israel	Master thesis	URL	n=48, F=48, 19-55 yrs	Between-subjects	Digital art making, vs traditional art making with Oil pastels, free drawing, 45 min	HRV, RSA, CSI	Salivary cortisol	State-Trait Personality Inventory (STPI), SAM, Art-based intervention (ABI), FSS, Formal elements art therapy scale (FEATS)	Comparing digital vs traditional drawing	Both mediums were successful in regulating emotions	The traditional medium may create a more connected art-making experience	Single gender, no control group	Evaluating state stress
**Shai Haiblum-Itskovitch et al (2018)**	Israel	Frontiers in Psychology	https://doi.org/10.3389/fpsyg.2018.00968	n=50, F= 24, 22-44 yrs	Order randomizes	Drawing with pencil, oil-pastels, and gouache paint (10 min per each task)	HRV	None	Self-Report, SAM	Comparing the emotional and physiological responses to different art materials in fluidity	Drawing with gouache paint and oil-pastels improves positive mood.	Fluidity of the material is not the reason for the effectiveness of artwork. Oil-pastels results in unique emotional and physiological responses.	Small sample	Measuring emotional response to the art making

Table 3-Overview of studies including pain and biological measurements (no art therapy)

**Author (Year)**	**Country**	**Journal**	**DOI**	**Population (n, gender, age)**	**Study design**	**Pain/Emotion type & duration**	**Physiological marker**	**Biomarker**	**Emotion measurement**	**Aim of study**	**Main results**	**Conclusion**	**Limitations**	**Notes**
**Luis Felipe Bortoletto et al (2025)**	Brazil	SPIE	https://doi.org/10.1117/	n=37, F=13, 19-33	Randomized study	Stress-oriented (30 to 50 seconds)	fMRI, Cardiovascular activity	Blood CO2 level	None	Finding a paradigm to assess the body's reactivity to stress	Stress elicited bilateral activation in the frontier	Proposed protocol induces stress which is shown in	--	--

			12.3046399								frontal gyri, and altering the cardiovascular and blood CO2 level	fNIRS signals		
**Steven J. Nichols et al (2023)**	USA	NMR In Biomedicine	https://doi.org/10.1002/nbm.5088	n=15, F=15, 22-25yrs	Not specified	Pressure-based pain by using standard blood pressure cuff (10 second)	fMRS	None	Generalized Anxiety Disorder-7 (GAD-7), Patient Health Questionnaire-9 (PHQ-9), Prodromal Questionnaire–Brief Version (PQ-B), Alcohol Dependence Scale (ADS), Nicotine Dependence Scale for Adolescents (NDSS-A), Severity of Dependence Scale (SDS), Graded Chronic Pain Scale (GCPS), Neuropathic Pain Scale (NPS).	Investigating the neurometabolite level in the dorsal anterior cingulate cortex (dASS)and Primary Somatosensory Cortex (SI) during acute pain	Increasing in glutamate levels following acute pain	Meaningful changes in (dASS) gamma aminobutyric acid in response to pain stimulation	Small sample size, only female	--
**Jan Weber et al (2019)**	USA and Germany	Experimental Brain Research	https://doi.org/10.1007/s00221-019-05531-0	n=33, F=16, 23-44 yrs	Not specified	30 days of Isolation	EEG	Cortisol, Neurotrophic factors	PANAS-X, Cognitive test battery	Investigating the effect of short-term isolation on physiological and psychological parameters	Stress level increased, No further significant changes	30 days of isolation do not have a significant effect on brain activity and mood, just as the stress levels increased	Small sample size	--
**Adam Bibbey et al (2013)**	Netherlands	International Journal of Psychophysiology	http://dx.doi.org/10.1016/j.ijpsycho.2012.10.018	n=352, F=190, 57-60 yrs	Not specified	Acute psychological stress protocol comprising: a Stroop task, mirror tracking, and a speech task (5 min each)	Cardiovascular activity	Salivary cortisol	Using Big Five Inventory to assess neuroticism	Investigating the type of personality on stress reaction	Higher neuroticism scores, less agreeable and less open exhibited smaller cortisol and cardiovascular stress reactions	Negative personality disposition would be linked to diminished stress reactivity.	Small observed effect size	Investigating the role of personality on stress reaction
**Maureen Groer et al (2010)**	USA	JOEM	https://doi.org/10.1097/jom.0b	n=141, F=27 ,22-64 yrs	Randomized study	2 virtual reality scenarios for inducing stress: 1-motorcycle scenario (2 min) 2-	Respiratory rate, ECG, Skin temperature,	Saliva (Alpha amylase, Cortisol, IL-6, SIgA)	Perception of how stressful the experience (a single item scored on a 1 to 5 Likert scale)	Investigating the effect of a critical incident lethal force scenario on salivary biomarkers	“workplace” scenario rises the level of cortisol significantly	Virtual reality can produce stress	Analyzing stress perception	Investigating research on

			013e3181e129da			workplace scenario (6 min)	HR, and HRV, eye tracking						just through a one item question with a 5-point Likert scale	Police officers
**Andrew STEPTOE et al (2001)**	UK	Clinical Science	https://doi.org/10.1042/cs1010185	n=20, F=12, 25-51yrs	Not specified	Mental stress including 2 computer based tasks (5 min each)	HR, Blood Pressure	Saliva, TNF-*a,* IL-6, and IL-1Ra	Behavioral performance of the tasks	investigate whether acute psychological stress leads to changes in circulating pro-inflammatory cytokines	significant increases in IL-6 and IL-1Ra concentrations in the stress group	inflammatory cytokines respond to acute mental stress in humans with delayed increases, and suggest that individual differences in cytokine responses are associated with sympathetic reactivity.	--	--
