# Supplementary figures and images for: Art therapy and emotional pain: a scoping review of physiological and biological measures
Source: Front Hum Neurosci. 2026 Mar 11;20:1736930. doi: 10.3389/fnhum.2026.1736930 (PMC13013050; doi:10.3389/fnhum.2026.1736930)

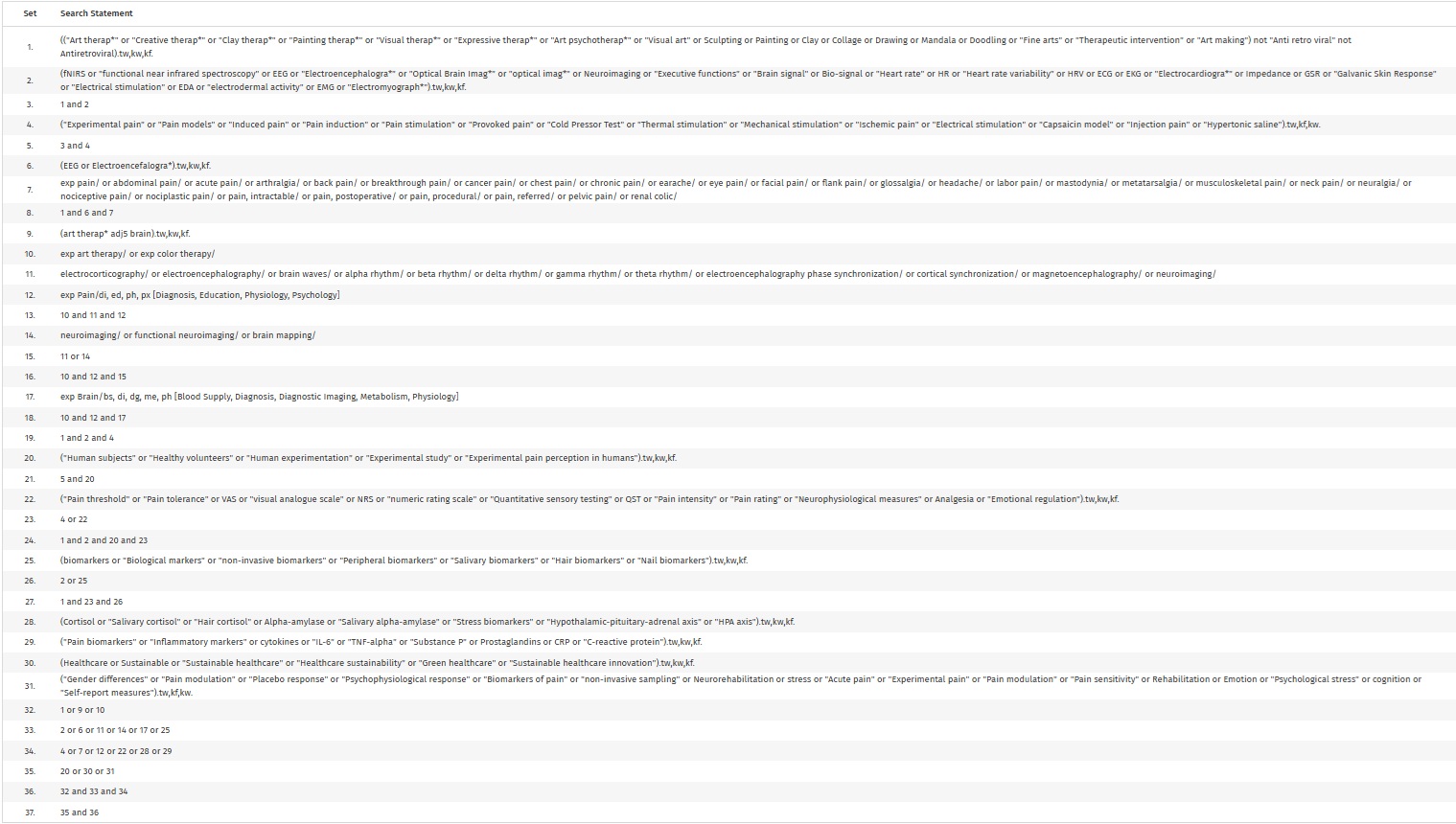

Supplement: Supplementary file 2 [file Image_1.jpg]
